# Multiple roles for a mitochondrial enzyme

**DOI:** 10.7554/eLife.107882

**Published:** 2025-06-16

**Authors:** Xicong Tang, Hongyu Qiu

**Affiliations:** 1 Cardiovascular Translational Research Center, College of Medicine-Phoenix, University of Arizona Phoenix United States; 2 Clinical Translational Sciences (CTS) and Bio5 Institution, University of Arizona Phoenix United States

**Keywords:** aging, arginase, heart, inflammation, fibrosis, macrophages, Human, Mouse, Rat

## Abstract

The enzyme arginase-II has an important role in cardiac aging, and blocking it could help hearts stay young longer.

**Related research article** Potenza DM, Cheng X, Ajalbert G, Brenna A, Giraud MN, Frobert A, Cook S, Mertz KD, Yang Z, Ming XF. 2024. Cell-autonomous and non-cell-autonomous effects of arginase-II on cardiac aging. *eLife* **13**:RP94794. doi: 10.7554/eLife.94794.

Aging is a natural biological process that causes structural and functional changes in many organs. In the heart these changes mean that its ability to pump blood declines with age, which can reduce blood flow to the entire body. Heart or cardiac aging is marked by several pathological changes, including the death of heart muscle cells (cardiomyocyte apoptosis), the accumulation of scar tissue, and reduced energy production due to mitochondrial impairment (Alibhai and Li, 2024; Derumeaux et al., 2024). Over time, these changes can reduce the heart’s pumping efficiency, which can lead to cardiac dysfunction or even heart failure (Strait and Lakatta, 2012). Aging also increases the risk of heart diseases and makes the heart more vulnerable to ischemic injury (North and Sinclair, 2012). However, the molecular mechanisms behind these changes are still not fully understood, which has hindered the development of effective therapies.

Now, in eLife, Duilio Potenza, Zhihong Yang, Xiu-Fen Ming and colleagues at the University of Fribourg report the results of experiments on mice which show that a mitochondrial enzyme called arginase-II (Arg-II; Caldwell et al., 2018) has a critical role in cardiac aging (Potenza et al., 2024). In particular, they show that the loss of Arg-II protects the heart against aging.

Using genetically modified mice that lack the Arg-II gene, Potenza et al. found significant protection against age-related cardiac changes. These mice showed reduced cardiomyocyte apoptosis, less accumulation of scar tissue (fibrosis), lower heart inflammation, and a reduction in endothelial-to-mesenchymal transitions. Additionally, these mice showed better resistance to ischemic injury. These observations suggest that Arg-II plays a key role in the development of age-related heart damage, making it a strong therapeutic target. This study also aligns with previous work by the same group showing that deletion of Arg-II decelerates the aging process and extends mouse lifespan (Xiong et al., 2017).

Somewhat surprisingly, although Arg-II contributes to cardiac aging, the researchers also found that it is not expressed in cardiomyocytes in either aged mice or aged humans, contrary to prior assumptions. Instead, Arg-II is upregulated in other cell types, such as macrophages, fibroblasts, and endothelial cells. This suggests Arg-II may affect heart function in an indirect, yet influential, way during the aging process.

Aging is often associated with chronic, low-grade inflammation, also known as inflammaging (Li et al., 2023), which can lead to the accumulation of immune cells and pro-inflammatory molecules within the heart. This accumulation can, in turn, affect the structure and function of the heart, and contribute to cardiac aging (Müller and Di Benedetto, 2024; Liberale et al., 2022; Goldstein and Abdel-Latif, 2023), but the details of this process are not completely understood.

The study of Potenza et al. found that Arg-II increases a key inflammatory molecule, IL-1β, especially in macrophages (Figure 1). IL-1β then affects cardiomyocytes, fibroblasts, and endothelial cells, promoting apoptosis, fibrosis, and endothelial dysfunction. Notably, when Arg-II was deleted, levels of IL-1β went down, and the harmful effects on heart cells were reduced. As a result, heart muscle cells stopped dying, fibrosis slowed down, and endothelial cells remained healthier. In addition to highlighting IL-1β as a major link between inflammation and cardiac aging, these findings show that it is also a potential therapeutic target.

**Figure 1.**
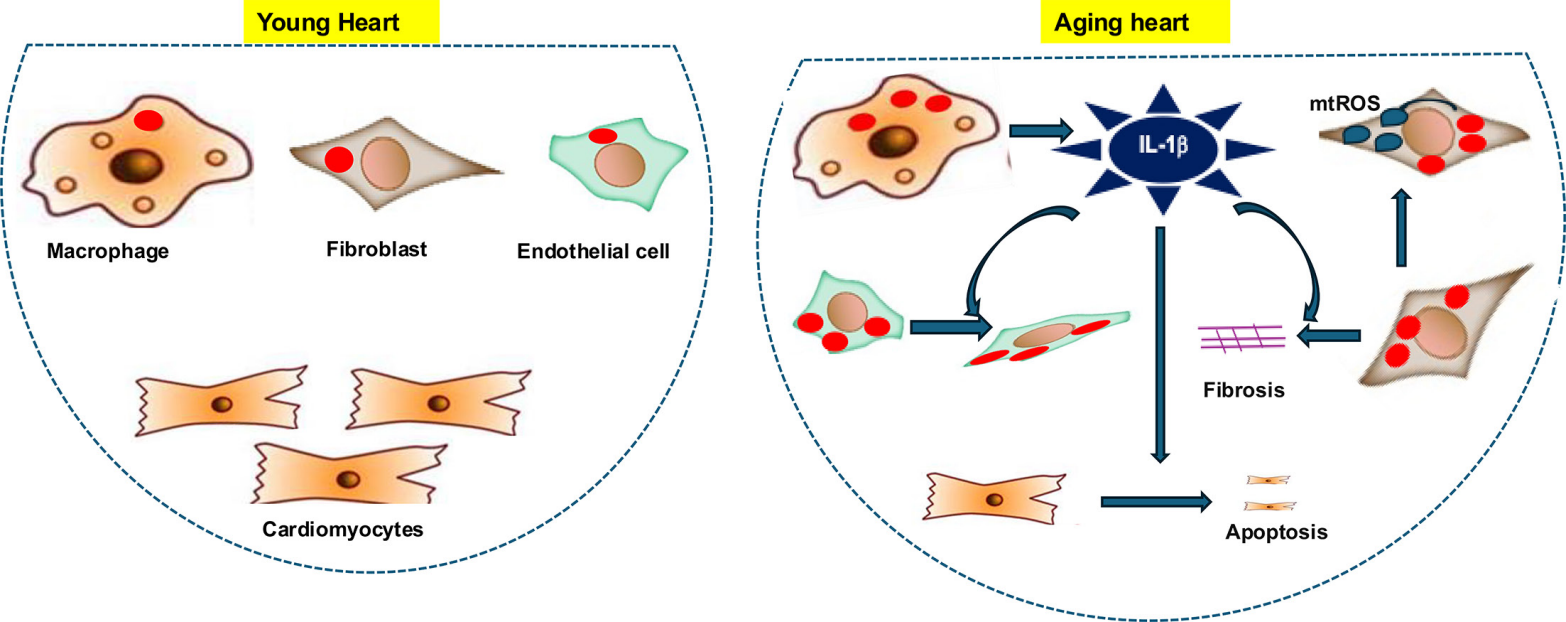
Arg-II as a driver of cardiac aging and a potential therapeutic target. In the young heart (left), the enzyme Arg-II (red ovals) is mainly found in cells other than cardiomyocytes, such as macrophages, fibroblasts, and endothelial cells. The levels of Arg-II also increase with age. In aging hearts (right), Arg-II in macrophages promotes the release of the inflammatory molecule IL-1β (dark blue). This leads to cardiomyocyte apoptosis, fibrosis (due to excess collagen production), and an increase in the number of endothelial cells transforming into mesenchymal cells. Arg-II also increases the production of harmful reactive oxygen species (mtROS) in fibroblasts. Therefore, blocking Arg-II or its downstream effects (such as IL-1β or reactive oxygen species) could reduce damage to the heart and slow cardiac aging.

Besides showing that Arg-II promotes inflammation, Potenza et al. showed that it also boosts the production of harmful reactive oxygen species in the mitochondria of the fibroblasts. This process, which is not mediated by IL-1β, further activates fibroblasts and causes more fibrosis, indicating that Arg-II acts in different ways in different cells. Thus, Arg-II contributes to cardiac aging through both immune-mediated and cell-intrinsic mechanisms.

The study points to several potential treatments: (i) blocking Arg-II to protect the heart from aging; (ii) correcting macrophage dysfunction, possibly using cell-based therapies, to slow cardiac aging; (iii) blocking downstream mediators, such as IL-1β or reactive oxygen species, to reduce cell death, inflammation and fibrosis. By uncovering the link between Arg-II and these pathways, the study opens the door to more precise treatments for heart disease in the elderly.

The work of Potenza et al. has the potential change how we think about cardiac aging by redefining the role of Arg-II in this process. It shifts the focus away from heart muscle cells and highlights the critical influence of immune cells and other types of cells (such as macrophages, fibroblasts, and endothelial cells). It also reinforces the idea that chronic inflammation is a key driver of cardiac functional decline with age, and suggests that blocking Arg-II-mediated inflammation may offer promising strategies to prevent, or at least slow down, the heart aging process.

Further research is needed to better understand how Arg-II is upregulated in aging cells and how its effects vary between cell types. The development of cell-specific therapies will be crucial. Additionally, translating these findings from animal models to humans remains a key challenge. Nonetheless, targeting Arg-II and inflammation holds great potential for promoting healthier hearts in aging individuals.
